# Which assisted reproductive technology (ART) treatment strategy is the most clinically and cost-effective for women of advanced maternal age: a Markov model

**DOI:** 10.1186/s12913-022-08485-2

**Published:** 2022-09-23

**Authors:** Evelyn Lee, Jinhui Zhang

**Affiliations:** 1 Centre for Economic Impacts of Genomic Medicine, Macquarie Business School, Macquarie University, Sydney, NSW Australia; 2 Department of Actuarial Studies and Business Analytics, Macquarie Business School, Macquarie University, Sydney, NSW Australia

**Keywords:** Assisted reproductive technologies (ART), Preimplantation genetic screening for aneuploidy, Oocytes cryopreservation, Donated oocytes, Cost-effectiveness analysis, Markov, Decision-analytic model

## Abstract

**Objective:**

To evaluate the clinical and cost-effectiveness of preimplantation genetic testing for aneuploidy, social freezing, donor and autologous assisted reproductive technology (ART) treatment strategies for women aged 35–45 following 6–12 months of infertility.

**Methods:**

Four Markov decision-analytic models comprising: (i) Preimplantation genetic testing for aneuploidy (PGT-A); (ii) autologous ART from age 40 using oocytes cryopreserved at age 32 (social freezing); (iii) ART using donated oocytes (donor ART); (iv) standard autologous ART treatment (standard care) were developed for a hypothetical cohort of 35 to 45 years old ART naïve women with 6–12 months of infertility. Input probabilities for key parameters including live birth rates were obtained from the available literature. Deterministic and probabilistic sensitivity analyses were conducted to address uncertainty in estimating the parameters and around the model’s assumptions. Cost effectiveness was assessed from both societal and patient perspectives .

**Result(s):**

For infertile women at age 40 and above, social freezing is the most cost-saving strategy with the highest chance of a cumulative live birth at a lowest cost from a societal perspective. PGT-A and donor ART were associated with higher treatment costs and cumulative live-birth rates compared with the autologous ART. Among the four ART strategies, standard autologous ART has the lowest cumulative live birth rate of 45% at age 35 and decreasing to 1.6% by age 45 years. At a willingness-to-pay threshold of Australian dollars (A$)50,000, our model shows all alternative treatment strategies –PGT-A, social freezing and donor ART have a higher probability of being cost-effective compared to the standard autologous ART treatment. However, higher out-of-pocket expenditure may impede their access to these alternate strategies.

**Conclusion:**

Given current evidence, all alternate strategies have a higher probability of being cost-effective compared to the standard autologous ART treatment. Whether this represents value for money depends on societal and individual’s willingness-to-pay for children conceived with ART treatment.

**Supplementary Information:**

The online version contains supplementary material available at 10.1186/s12913-022-08485-2.

## Introduction

Assisted reproductive technology (ART) has revolutionised the treatment of infertility over the last three decades, with more than 2.5 million ART cycles performed annually and the birth of over 6.5 million children conceived worldwide to date [1]. Despite the mainstream acceptance of ART treatment and improvements in overall success rates, women of advanced maternal age (AMA) defined as ≥35 years of age, remain a challenge for fertility physician [2, 3]. According to the latest report from the Centers for Disease Control and Prevention on ART cycles performed in the United States (US) in 2018, the live-birth rate per initiated cycle for women aged 41–42 years was 13.2% compared to 55.1% for women aged below 35 years [4].

Although hormonal, uterine and oocyte factors play a role in the relatively poor success rates in women of AMA [5, 6], chromosomal abnormalities (i.e., aneuploidy) associated with advanced maternal age are largely responsible for ART treatment failure [7, 8]. It has been reported that by age 40, 80% of oocytes are already aneuploid [9].

As clinical and laboratory techniques for ART continue to evolve and improve, a number of treatment strategies that are often more costly than those conventionally available have been proposed to improve success rates in older women, but no studies have systematically compared the clinical and cost-effectiveness of contemporary approaches in this patient group.

Three treatment strategies that are increasingly used to augment ART in older women are preimplantation genetic testing for aneuploidy (PGT-A), cryopreservation of autologous oocytes at a younger age for potential later use (‘social freezing’), and the use of donated oocytes (donor ART).

Preimplantation genetic testing for aneuploidy in all 24 chromosomes using advanced genetic techniques has been shown in observational studies and randomised trials to improve implantation, clinical pregnancy and live-birth rates in women of AMA [2, 10–12]. For example, Lee and colleagues reported older women (mean age 40.1 years) who used PGT-A and blastomere aCGH (array comparative genomic hybridization) analysis for blastocyst transfer achieved a higher live birth rate using fewer ART cycle compared with standard morphologic embryo selection at the blastocyst stage (14.5% vs 9.1% respectively) [2].

Social freezing, where autologous oocytes are collected and cryopreserved at a younger age for potential future use, is increasingly used as an option to negate the risk of age-related fertility loss [13]. In a recent retrospective US study of 921 women who vitrified their oocytes between 2006 and 2020, women who cryopreserved their oocytes before aged 38 achieved a higher cumulative live birth rate (CLBR) (38.9%) than those who planned their oocytes cryopreservation after age 38 (25%) [14]. This is also shown in a previous study by Cobo and colleagues who reported CLBR increased for every additional oocytes in women below 36 years old but this reached a plateau for older women ≥ age 36 [15].

The most traditional approach to improving ART success in women of AMA is the use of donated oocytes from younger women. Rates of donor ART continue to increase in the US, with 10,801 donor cycles performed in 2000 to around 24,000 in 2016, accounting for 7.5% of all ART cycles and 10.7% of cycles in women over aged 40 years and over [16]. Success rates following donor ART remain relatively good in women aged over 40 years, with more than one in four cycles resulting in a term singleton live-birth [17].

The aim of this study is to use a decision-analytical Markov model to assess the clinical and cost-effectiveness of four main ART strategies used in women of AMA. The study seeks to answer a common clinical question faced by the physician and patient: ‘Which ART strategy is the most clinically and cost-effective for women aged 35 years and over following 6-12 months of infertility?’

## Material and methods

### Model structure and strategies

A Markov model was developed to represent each of the four main treatment strategies for ART naïve women aged from 35 to 45 years following 6–12 months of infertility. All Markov models were configured individually to represent the likely clinical pathway in terms of OPU procedure, cancelled cycle and returning for subsequent fresh or FET cycles at each individual age from 35 to 45 years. The schematic representation of the four main treatment strategies is summarised below and presented in Supplementary Fig. 1.

### ART strategies

1. Standard autologous ART: this treatment strategy involves undertaking two ‘complete autologous’ ART cycles. A ‘complete autologous’ cycle in the standard strategy is defined as a fresh autologous cycle followed by two subsequent FET cycles resulting from one episode of ovarian stimulation. The model started with ART naïve women aged between 35 and 45years commencing treatment by entering the ‘fresh cycle’ state where she could either proceed to an oocyte pick-up (OPU) or the cycle is cancelled (i.e., due to poor response or hyper-stimulation). If the cycle was cancelled, she could either end her treatment or undertake a second fresh cycle. If no live birth was achieved after her first complete cycle, she could end her treatment or commence a second fresh cycle. The strategy pathway ends when two ‘complete autologous’ cycles have been undertaken.
2. PGT-A: this strategy involves undertaking two ‘complete autologous’ cycles with PGT-A. In this strategy, a ‘complete PGT-A autologous cycle’ is defined as a fresh autologous cycle followed by one subsequent FET cycle resulting from one episode of ovarian stimulation. The model started with an ART naïve woman aged between 35 and 45 years entering the model in a ‘fresh cycle’ state where she could either proceed to an OPU procedure or cancelled cycle. If the initial fresh cycle was cancelled, the woman could either end her treatment or undertake a second fresh cycle. If no live birth was achieved after her first complete cycle, she could end her treatment or commence a second fresh cycle. The protocol of PGT-A is assumed to be blastomere aCGH to select euploid embryos for transfer during fresh and FET cycles.
3. Social freezing: this strategy involves oocyte cryopreservation at age 32 with women returning between age 40 and 45 years for ART using their stored oocytes. In this strategy, the model started with a 32-year-old woman entering the ‘fresh cycle’ leading to an OPU procedure or cancelled cycle. If the fresh cycle was cancelled, the women could either end her treatment or undertake another fresh cycle. The model assumes that two OPU procedures will retrieve sufficient oocytes for two autologous FET cycles for women returning at aged between 40 and 45 years. The strategy pathway ends when two FET cycles using vitrified oocytes have been undertaken.
4. Donor ART: this strategy started with an infertile ART naïve woman aged between 35 and 45 years entering the model to undertake two ‘complete autologous’ cycles followed by two donor ART cycles. In this strategy, a ‘complete autologous’ ART cycle refers to a fresh autologous cycle followed by two subsequent FET cycles resulting from one episode of ovarian stimulation. If the woman was unsuccessful in achieving a live birth after two ‘complete autologous’ cycles, she could either end her treatment or undertake up to two FET cycles using donated oocytes (donor ART cycles). The ‘donor ART’ strategy pathway ends after two ‘complete autologous’ cycles followed by two donor ART cycles have been undertaken.

### Health states

There were four health states in each strategy: ‘fresh cycle’, ‘frozen embryo transfer (FET)’, ‘live birth’ and ‘end’. Women aged between 35 and 45 years were modelled starting in one of four treatment strategies and transitioning to one of these health states: ‘fresh cycle’, 'FET'’, ‘live-birth’ and ‘end’ based on assigned age and health state-specific transition probabilities. For example, women aged 40 years who commenced ART autologous strategy at the ‘fresh cycle’ health state and did not achieve a live birth could end the treatment or start a new fresh cycle.

In all strategies, ‘live-birth’ and ‘end’ were absorbing states, meaning that women who entered these states cannot move to another health state. The model kept track of treatment costs and live-birth rates associated with each health state so that as the model simulated the cohorts progressing through each strategy, the expected mean costs, cumulative live-birth rate and cost-effectiveness were calculated. The cycle time of the Markov model was defined as one complete ART cycle (fresh and/or up to two FET cycles) with each treatment strategy completed within a year.

### Assumptions

Women can end from treatment when (i) a cycle is cancelled (e.g., due poor response or hyperstimulation) or (ii) a live birth is not achieved after embryo transfer. In the model, treatment strategy ends when (i) a live birth is achieved and (ii) treatment pathway is completed.

### Data sources

The model inputs are summarised in Table 1.

**Table 1:** Model inputs

	Probability estimates	Distribution	References
	Per Cycle		
**Initiated cycles reaching oocyte pick-up (OPU)**			
Age 35–39	0.93	Beta	[18]
Age 40–44	0.90	Beta	[18]
Age 45	0.85	Beta	[18]
**Progression to fresh cycle**			
Age 35–39	0.765	Beta	[18]
Age 40–44	0.707	Beta	[18]
Age 45	0.707	Beta	[18]
**Autologous ART**			
Cumulative live birth rate per OPU			
Age 35	0.325	Beta	[18]
36	0.286	Beta	[18]
37	0.249	Beta	[18]
38	0.213	Beta	[18]
39	0.179	Beta	[18]
40	0.160	Beta	[18]
41	0.142	Beta	[18]
42	0.107	Beta	[18]
43	0.074	Beta	[18]
44	0.043	Beta	[18]
45	0.014	Beta	[18]
**Social Freezing**			
Cumulative live birth rate			
Age 40	0.351	Beta	[18, 15]
41	0.338	Beta	[18, 15]
42	0.325	Beta	[18, 15]
43	0.313	Beta	[18, 15]
44	0.300	Beta	[18, 15]
45	0.287	Beta	[18, 15]
**PGT-A**			
Cumulative live birth rate			
Age 35	0.337	Beta	[2,11]
36	0.295	Beta	[2,11]
37	0.244	Beta	[2,11]
38	0.194	Beta	[2,11]
39	0.166	Beta	[2,11]
40	0.153	Beta	[2]
41	0.149	Beta	[2]
42	0.147	Beta	[2]
43	0.140	Beta	[2]
44	0.120	Beta	[2]
45	0.082	Beta	[2]
**Donor ART**			
Cumulative live birth rate			
Age 35	0.380	Beta	[18]
36	0.380	Beta	[18]
37	0.380	Beta	[18]
38	0.317	Beta	[18]
39	0.317	Beta	[18]
40	0.317	Beta	[19]
41	0.329	Beta	[19]
42	0.329	Beta	[19]
43	0.378	Beta	[19]
44	0.378	Beta	[19]
45	0.333	Beta	[19]

As live birth rates for different strategies (standard autologous ART, PGT-A, social freezing and donor ART) were summarised and reported by 5-year interval in the literature, logistic regression was used to obtain age-specific live birth rate. The regression formulas are presented in Appendix A (Supplementary Materials).

Unless otherwise stated, live-birth rates using autologous fresh, FET and donated ART cycles, OPU rate, cycle cancelation, transition probabilities and discontinuation rates were sourced from the Australian and New Zealand Assisted Reproduction Database (ANZARD) held at the National Perinatal Epidemiology and Statistics Unit of the University of New South Wales, Sydney [18]. ANZARD collects information on all ART treatment cycles undertaken in Australia and New Zealand, including the resulting treatment and pregnancy outcomes.

### PGT-A

The live-birth rates after PGT-A for women aged between 35 and 39 were taken from a US study that have utilised ART data from the CDC National ART Surveillance Systems for 2011–2012. [11] For aged between 40 and 45, CLBR per complete cycle with PGT-A were based on a cohort study on PGT-A using blastomere aCGH conducted on relatively good prognosis older women (mean age 40.1) [2]. In our model, we assume that there was a 10% reduction in the live-birth rate in the subsequent fresh cycle using PGT-A. Live-birth rates in FET cycles were assumed similar to estimates used in the fresh cycle.

### Donor ART

The live-birth rate of ART donor cycles for women aged between 35 and 39 was sourced from ANZARD. The donor programme in Australia and New Zealand accounted for about 5% of all ART cycles in the 5 years to 2014 [18]. For women aged between 40 and 45 years, live birth rates of ART donor cycles were taken from a population-based cohort study of 987 Australian women (mean age 41.4 years) who obtained donated oocytes between 2009 and 2016. In the model, we used a live-birth rate based on the mean age of oocyte donors of 32 years for all recipients [19].

### Social freezing

The live-birth rate following social freezing was sourced from the retrospective multicentre study by Cobo and colleagues [15] and ANZARD [18]. In the study by Cobo and colleagues, the authors reported on average, 12.7 oocytes were retrieved and 9.7 metaphase II (MII) oocytes were vitrified among the 1,382 women (mean age of 37.7 years) who vitrified their oocytes to prevent age-related infertility. The model assumes women undertook two OPU to retrieve an average of 12.7 oocytes per woman for a cumulative live birth [20].

### Standard autologous ART

All age and transition probabilities and live-birth rates were sourced from ANZARD.

### Costs

A summary of cost estimates of resources consumed in the delivery of ART services is listed in Table 2. Costs reflected the direct healthcare costs of ART treatment including public costs covered by Medicare (Australia’s universal health insurance scheme), private health insurers and patient out-of-pockets (OOP) expenses.

**Table 2:** Cost estimates

Types of treatment	Distribution	Medicare rebate^**a**^ (USD)	Out-of-pocket costs (USD)
Cost of cancelled cycle^b^	Gamma	1,463	1,909
Cost of complete fresh and FET autologous ART cycle^c^	Gamma	6,522	4,325
Cost of FET cycle	Gamma	626	1,023
Cost of PGT-A^d^	Gamma	–	1,813
Cost of complete donor ART cycle^e^	Gamma	–	11,720
Cost of frozen oocytes storage per year	Gamma	–	260

^a^Almost all autologous ART treatment cycles are subsidised through the Australian Government’s universal insurance scheme, Medicare where women receive partial reimbursement of all ‘medically necessary’ ART procedures

^b^Cancelled cycle refers to a ‘superovulated’ cycle that is cancelled prior to oocyte retrieval. Cost includes planning and management fee, semen preparation and pharmaceutical drugs

^c^Cost includes planning and management fee, oocyte retrieval, ultrasound examination, counselling, ovulation monitoring service, and preparation of semen, fresh embryo transfer and pharmaceutical drugs

^d^Cost includes screening of developing embryo(s) from one stimulated ART cycle using blastomere aCGH for transfer during fresh and FET cycles

^e^Cost includes planning and management, use of anonymous donor oocytes, ultrasound examination, counselling, and embryo transfer

All costs were rounded off to the nearest integer. USD: 2019 United States Dollars; PGT-A: Preimplantation genetic testing for aneuploidy; FET: Frozen embryo transfer; ART: Assisted reproductive technology

Unit cost charged by providers for partial and complete ART treatment cycles and procedures were based on a review of clinic fee schedules published online by Australian fertility clinics (64 of 84 individual clinics published their schedule fees). The difference between the actual fee charged by providers and the Medicare are borne by the patients. As most adjunct ART procedures and techniques such as PGT-A and oocytes freezing do not attract a subsidy through Medicare, patients pay the full amount out of their own pocket.

For example, on average, the out of pocket expenditure for a patient undertaking social oocytes strategy includes ovarian stimulation medication, OPUs to retrieve oocyte for freezing and storage ($9305) and then returning for up to two cycles of frozen embryo transfers using stored oocytes ($3,300) was $12,605. The spending data were normalized to 2019 Australian dollar value, using the Australian Institute of Health and Welfare inflation rate [21].

### Perspectives

The economic evaluation was undertaken from a societal perspective in which all direct costs of treatment, regardless of who pays or receives the benefits, are incorporated into the analysis, as well as from a patient perspective in which only out-of-pocket costs were included. The purchasing power parity (PPP) conversion rate used in this study was US $1 is equal to AU $1.41 [22].

### Clinical and cost-effectiveness analysis

For this analysis, we modelled the age-specific clinical and cost-effectiveness for all four main treatment strategies (PGT-A, social freezing, donor ART and standard autologous ART). The primary clinical outcome measure is CLBR per strategy. The economic evaluation measures are the mean costs and CLBR per strategy, and the incremental cost-effectiveness ratio (ICER). The ICER is calculated as the ratio of incremental costs and outcomes between alternate and the reference strategy and reflects the extra cost needed to obtain an additional live birth by adopting the alternative strategy (i.e., PGT-A, Donor ART and social freezing) over the standard autologous ART strategy [23].

### Sensitivity analysis

We conducted probabilistic sensitivity analyses (PSA) for the base case results for all four main treatment strategies (PGT-A, social freezing and donor ART versus standard autologous ART). The results of the PSA are presented in a cost-effectiveness (CE) plane to determine the effect on the ICER of the joint uncertainty in model parameters by repeating the cost-effectiveness analysis 10,000 times. Beta distributions were fitted to reflect the uncertainty in the effects, and gamma distributions were fitted to reflect uncertainty in costs [23]. For each of the 10,000 samples analysed, a parameter value and cost were randomly selected from the assigned probability distributions. The 2.5th and 97.5th percentile were calculated, which indicated the borders of the 95% confidence interval.

All economic and sensitivity analysis were performed in Excel and STATA software version 14 (Statacorp).

## Results

Table 2 summarises the results of the age-specific clinical and cost-effectiveness results for ART naïve women commencing treatment between aged 35 and 45 years.

### Clinical effectiveness

**Fig. 1:**
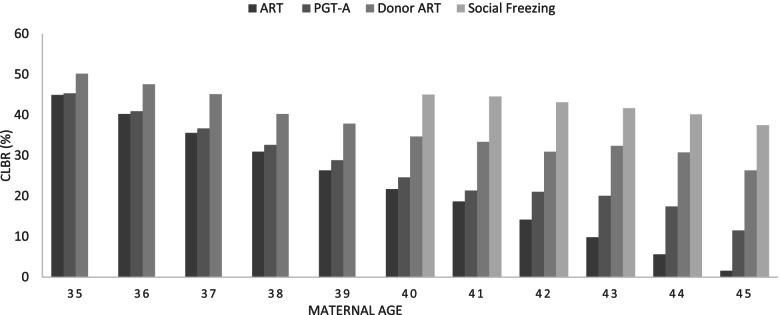
Cumulative live birth rate by treatment strategy and maternal age. Note: Social freezing strategy involves women cryopreserved her oocyte cryopreservation at age 32 and returning at age 40 or above for ART for thaw cycles

All alternative strategies were associated with a higher CLBR compared with standard autologous ART strategy. For women aged between 35 and 40, donor ART strategy has the highest CBLR, and after age 40, a strategy of social freezing was more than twice as clinically effective in achieving CLBR compared with standard autologous ART strategy. Among the four ART strategies, standard autologous ART has the lowest CLBR with 45% at age 35 and decreasing to 1.6% by age 45 years. Figure 1 shows the CLBR by treatment strategy and maternal age.

### Cost-effectiveness

The expected mean cost, live-birth rates and ICER of the three alternative strategies – PGT-A, social freezing and donor ART– were compared with the standard autologous ART strategy, from a societal perspective (Supplementary Fig. 2).

For women aged between 35 and 40, donor ART was the most expensive treatment strategy but clinically more effective compared to the standard autologous ART, generating an ICER ranging between $26,240 and $17,732 respectively for one additional live birth. However, for women aged 40 and above, social freezing is a cost-saving strategy. In other words, for women between 41 and 45 years of age, oocytes cryopreservation at a younger age yields the highest chance of cumulative live birth at a lower cost compared to the standard autologous ART, and other alternate strategies in the model (Table 3).

**Table 3:** Incremental cost-effectiveness ratio of alternate treatment strategy relative to standard autologous ART treatment

Age	PGT-A	Donor ART	Social Oocytes
	Mean ICER (USD)	Mean ICER (USD)	Mean ICER (USD)
35	17,790 (−133,529 to 124,370)	26,240 (20,964 to 34,639)	–
36	7,997 (− 113,502 to 103,557)	19,674 (16,377 to 242,56)	–
37	3,282 (− 93,270 to 85,661)	15,850 (13,665 to 18,757)	–
38	250 (− 61,855 to 55,078)	23,244 (19,920 to 28,036)	–
39	More effective and cost saving	19,504 (17,056 to 22,758)	–
40	17,976 (4742 to 127,836)	17,732 (15,832 to 20,125)	More effective and cost saving
41	21,256 (−66,525 to 168,998)	13,422 (12,091 to 15,071)	More effective and cost saving
42	5,269 (1926 to 13,031)	12,189 (11,067 to 13,557)	More effective and cost saving
43	1,947 (101 to 5,107)	10,086 ( 9321 to10,990 )	More effective and cost saving
44	1,111 (− 366 to 3,513)	9,918 ( 9,225 to 10,758to )	More effective and cost saving
45	2,246 (537 to 5,683)	12,718 (11,899 to 13,900)	More effective and cost saving

^a^Social freezing strategy involves women cryopreserved her oocyte cryopreservation at age 32 and returning at age 40 or above for ART for thaw cycles

95% confidential interval in the parenthesis was derived from bootstrapped of 10,000 simulations USD: United States Dollars; ICER: Incremental cost-effectiveness ratio; PGT-A: preimplantation genetic testing for aneuploidy;ART: Assisted reproductive technology

Although PGT-A strategy was associated with a higher ICER when compared to the standard autologous ART strategy, the additional treatment cost to achieve one live birth with PGT-A decreases with an increase of age. Specifically, on average, the additional treatment costs to achieve to a live birth with PGT-A was $17,790 at age 35 and this decreases to $2,246 by aged 45 as PGT-A strategy was clinically more effective compared to the standard autologous ART strategy.

### Patient’s perspective

**Fig. 2:**
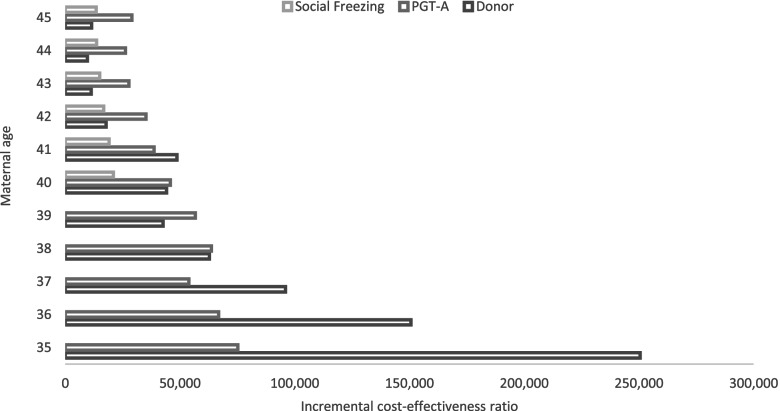
Incremental cost-effectiveness ratio (patient perspective). **Note**: Patient perspective refers to patient’s out of pocket expenditure for different treatment strategy relative to standard ART strategy to achieve a live birth

In addition to the societal perspective, we repeated the cost-effectiveness analysis based on out-of-pocket costs for infertility treatments. In Australia, patients pay out-of-pocket cost of approximately one-third of the cost of an autologous ART cycle (fresh and FET) plus all direct costs associated with alternative strategies (i.e., PGT-A, social freezing, OPU to retrieve and store oocytes for social freezing purposes and donor ART cycles). The results show that the standard autologous ART was associated with the lowest out-of-pocket costs to achieve a live birth for women aged between 35 and 45 years as more than half of direct treatment costs of standard ART services are reimbursed through Medicare. Due to a higher CLBR, social freezing incurred the least out-of-pocket expenditure to achieve a live birth compared to other alternate strategies for women aged ≥40 years. This is followed by PGT-A strategy which incurred the least out-of-pocket expenditure to achieve a live-birth for women aged after 42 years (ranging from $11,321 ─$11,495) in the model. Our findings showed a different relative order of cost effectiveness outcomes when assessed from a patient perspective as OOP expenses were higher with the social freezing strategy (Fig. 2).

### Probabilistic Sensitivity analysis

**Fig. 3:**
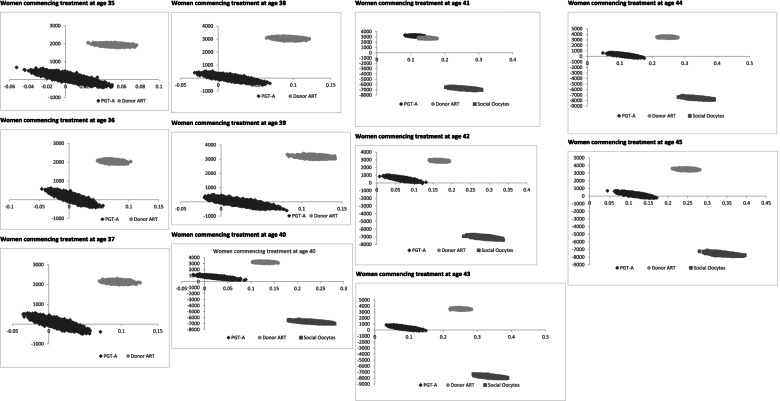
Incremental cost-effectiveness ratio scatterplots by maternal age

To assess the impact of parameter uncertainty in the model, a probabilistic sensitivity analysis was performed. In total, Markov Carlo simulation of 10,000 samples were conducted using variable values, sampled from the probability distribution around the variables’ mean values. Considering a threshold ICER of $50,000, other alternative strategies –PGT-A and donor ART were more costly and clinically more effective compared to standard autologous ART strategy for women aged between 35 and 45. Although social freezing was a cost-saving strategy, the high OOP expenditure may become a financial barrier to access treatment to cryopreserve their oocytes at a younger age to improve their chance of a cumulative live birth after aged 40 years. Age-specific incremental cost-effectiveness scatter plots illustrating the incremental costs with the alternate strategies plotted on the *y* -axis and the incremental effectiveness (i.e., additional live births) plotted on the *x* -axis are shown in Fig. 3.

## Discussion

This study addresses a common clinical and policy question regarding which ART treatment approach offers infertile women of advanced maternal age, the most clinically and cost-effective strategy to achieve parenthood.

From a societal perspective, the study found that for such women, social freezing was a cost-saving strategy. Although the other alternative strategies – PGT-A and donor ART– were associated with higher treatment costs, cumulative live birth were higher compared to the autologous ART. Using a cost-effectiveness threshold of US$50,000 per additional live birth, the three alternate treatment strategies had the highest probability of being cost-effective (or saving) relative to the reference ART strategy.

Our results are consistent with findings from recent studies which found a strategy of social freezing leads to higher live-birth rates per cycle compared with morphology-based assessment of embryos in older women [14, 20]. For example, using a decision tree model,, Devine and colleagues predicted that oocyte cryopreservation would decrease cost per live birth from US$55,060 to US$39,946 (and increase the odds of live birth from 42% to 62%) for women who freeze their oocytes at age 35 years and defer pregnancy attempts until age 40 years [20]. Klüber C and colleagues found that while oocyte freezing at aged between 25 and 38 yielded a higher CLBR at aged 40 (16.1 ─ 19.9 additional live births), OOP expenses (€34,959 for a live birth) were significantly higher compared to natural conception or standard ART treatment using fresh oocytes. The study also showed that return rate (i.e., women who returned to use their frozen oocytes) affected cost-effectiveness outcomes [24].

Although there is a paucity of data on the cost-effectiveness of PGT-A, a recently published cost-analysis conducted alongside a RCT in older women, found a higher mean cost per live-birth with PGT-A than conventional ART cycles with morphologic embryo selection (€23,895 vs €21,968, respectively) [12]. However, it is anticipated that as the technology matures, the cost of PGT-A will decrease and become more affordable for patients and lead to fewer overall cycles needed to achieve a baby [25]. Given that every additional cycle of IVF in the US costs patients around $15,000 and only five states mandate third party insurance coverage of ART, the cost-effectiveness of any strategy that reduced the cumulative number of cycles needed, including PGT-A, will improve over time [25] .

Even in countries that have supportive funding arrangements for ART, such as Australia, embryo selection techniques, donor cycles, and social freezing are not publicly funded. Indeed, previous studies found public were divided over public healthcare funding for non-medical oocyte preservation. For example, a recent Australian study reported that while a large majority of women supported public funding for embryo freezing for medical reason, less than half (42%) indicated their support for funding to non-medical social embryo freezing [26]. The divided view over public funding of alternate treatment is an important consideration as affordability is a significant determinant of equity and accessibility to fertility treatment. This also can have a significant impact of the cost-effectiveness of a strategy, as shown in our study where the out-of-pocket cost for the alternate treatment strategies was almost more than twice the cost of undertaking autologous ART treatment.

This is also found in a US internet-based survey of general population where women reported their willingness to pay an average of $3,811.55 to retrieve and store their oocytes for later use. However, when the cost of social freezing was increased to $10,000, women were willing to pay for the procedure only if the chance of achieving a live birth was at least 50%, suggesting that for an alternative treatment strategy to become mainstream, it needs to be more cost-effective than standard ART treatment [27].

To our knowledge, this is the first study to assess the clinical and cost-effectiveness of alternate ART treatment strategies in women aged 35 and above. While an RCT provides the highest level of evidence, they are difficult to undertake in fertility treatment, and rarely follow up patients over multiple cycles to measure cumulative live birth rates. Few studies have compared cost-effectiveness between multiple interventions with most studies are of a single intervention [20, 28, 29]. Furthermore, we used the most contemporary evidence to inform our model parameters and undertook sensitivity analysis to assess the robustness of our results. In particular, the model was based on data from real-world setting of women who returned to use their stored oocytes that were vitrified to prevent age-related infertility [15]. This overcomes the limitation of previous cost studies which used live-birth rates of thawed cycles from infertile women treated at fertility clinics or oocyte-donated programmes to assess the relative clinical and cost-effectiveness between social freezing and autologous cycles [20,24,30,31]. This has been recognised as an important research gap in cost-effectiveness analysis of social freezing in a recent editorial commentary [32].

As clinical and laboratory techniques for ART continue to evolve and improve, clinicians and infertile couples are increasingly presented with treatment options that are often more costly than those conventionally available. Cost-effectiveness analysis provides a useful tool for policymakers to balance this trade-off between costs and outcomes and ensures that treatments provide value for money based on societal and individual’s willingness-to-pay for children conceived via ART treatment. This is an important consideration as newer ART procedures and techniques are almost universally not covered by either public or third-party insurance plans which could hinder access to these strategies for extending reproductive potential and improve pregnancy rates. However, beyond the economic value of alternative strategies, women undergoing ART treatment may place different values on treatment characteristics, such as safety and burden (side-effects), health safety of future children and the risk of adverse perinatal outcomes when deciding on treatment options [33].

This study has several limitations. First, it is a simulation and reliant upon the quality of its model inputs obtained from published literature. The data for the base population were mostly obtained from national registry (ANZARD) that represent the most robust data sources and reasonable input to assess the outcomes. Second, we assumed 100% return rate amongst women who cryopreserved their oocytes which may affect cost-effectiveness outcomes. Previous studies have reported rates of return that range between 3 and 26% [34–36]. Finally, while it is acknowledged that cost-effectiveness analysis is unable to inform the level of resources that need to be invested on healthcare, they provide useful information that can be used to ensure that those limited resources, are used as effectively as possible to improve health wellbeing [37].

Given the continued trend to later childbearing and the commensurate need for fertility treatment, considerations of both the clinical and cost-effectiveness of alternative approaches to treatment are important to inform funding decisions and treatment choices.

## Supplementary Information

**Additional file 1: Supplementary Figure 1.** Markov processes for the ART strategies in the model. **Supplementary Figure 2.** Mean treatment costs per cumulative live birth by treatment strategy and maternal age. **Appendix.**

## Data Availability

All data generated or analysed during this study are included in this published article.
