# Supplementary material for: Which assisted reproductive technology (ART) treatment strategy is the most clinically and cost-effective for women of advanced maternal age: a Markov model
Source: BMC Health Serv Res. 2022 Sep 23;22:1197. doi: 10.1186/s12913-022-08485-2 (PMC9508737; doi:10.1186/s12913-022-08485-2)
Supplement: Supplementary file 1 — Additional file 1: Supplementary Figure 1. Markov processes for the ART strategies in the model. Supplementary Figure 2. Mean treatment costs per cumulative live birth by treatment strategy and maternal age. Appendix. [file 12913_2022_8485_MOESM1_ESM.docx]

**Supplementary Figure 1: Markov processes for the ART strategies in the model**


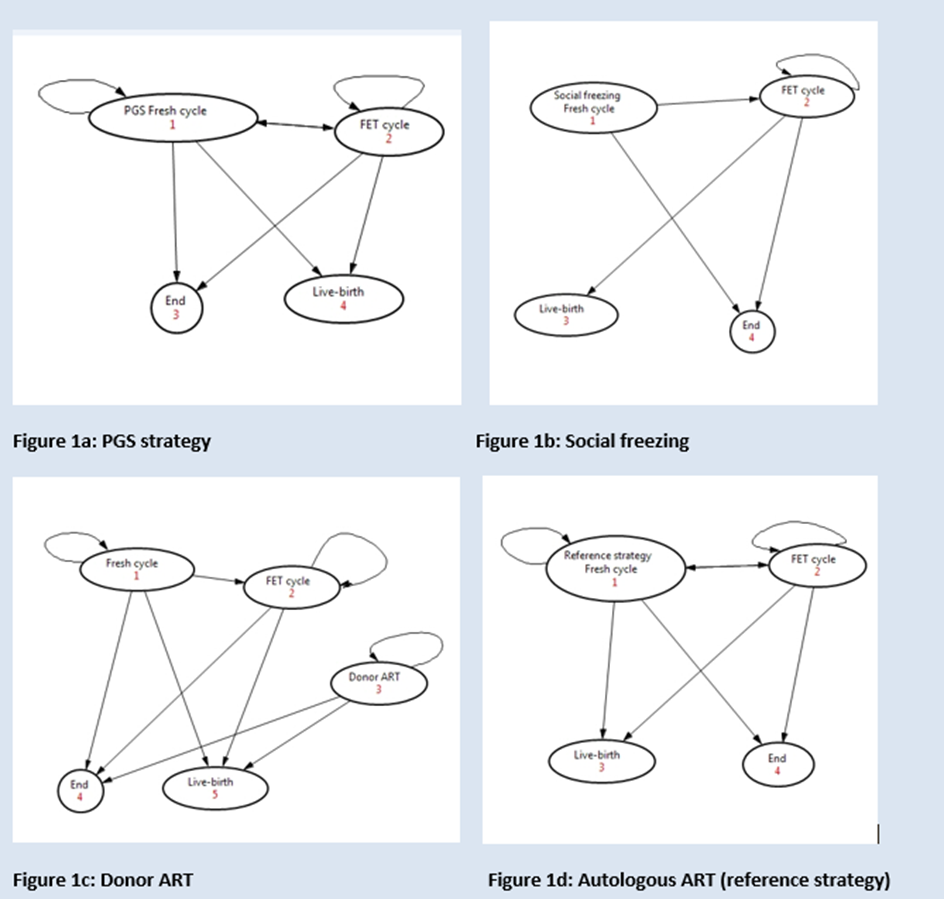


**Note:** The schematic representation of the four main treatment strategies for ART naïve women aged from 35 to 45 years following 6-12 months of infertility. **In Figure 1a,** the PGS strategy involves undertaking two ‘complete autologous’ cycles with PGT-A; **Figure 1b** (social freezing strategy) involves oocyte cryopreservation at age 32 with women returning between age 40 and 45 years for ART using their stored oocytes. **Figure 1c** (Donor ART) involves undertaking two ‘complete autologous’ cycles followed by two donor ART cycles. **Figure 1d** (standard autologous ART) involves undertaking two ‘complete autologous’ ART cycles where a ‘complete autologous’ cycle in the standard strategy is defined as a fresh autologous cycle followed by two subsequent FET cycles resulting from one episode of ovarian stimulation.

**Supplementary Figure 2: Mean treatment costs per cumulative live birth by treatment strategy and maternal age**

**Note:** For women aged 35– 40, donor ART was the most costly treatment strategy but clinically more effective to achieve a live birth compared to the standard treatment (autologous ART). For women aged 41-45, oocytes cryopreservation at a younger age (i.e., social oocytes strategy) yields the highest chance of cumulative live birth at a lower cost compared to the reference autologous strategy and other alternate strategies in the model.

**Appendix A:**

In this appendix we describe how the age-specific live birth rate can be obtained by the following formula:

First, we applied an ordinary least squares regression model (OLS) to the logarithm of the odds of live birth rate where live birth rate is treated as a dependent variable and the mid-point of the age group is regarded as the independent variable. We then use p to denote the live birth rate and define the odds of birth rate as p/(1-p).

Here, logistic regression estimates the logarithm of the odds as a linear combination of coefficients $\alpha$ and $\beta$, and age x: $\log\left( \frac{p}{1-p} \right)=\alpha+\beta x$. With the estimated $\alpha$ and $\beta$ coefficients values, we then find the estimated odds , $\log\left( \frac{\hat{p}}{1-\hat{p}} \right)=\alpha+\beta x$, and live birth rate, $\hat{p}=\frac{e^{\alpha+\beta x}}{1+e^{\alpha+\beta x}}$, for individual age.
